# Duane Retraction Syndrome With Mechanical and Innervational Upshoot and Secondary Superior Rectus Contracture: A Surgical Challenge

**DOI:** 10.7759/cureus.30470

**Published:** 2022-10-19

**Authors:** Jason Allan Seng Soon Cheah, Sangeetha Tharmathurai, Nor Akmal Bahari, Jamalia Rahmat, Julieana Muhammed

**Affiliations:** 1 Department of Ophthalmology and Visual Science, School of Medical Sciences, Universiti Sains Malaysia, Kelantan, MYS; 2 Ophthalmology, Hospital Kuala Lumpur, Kuala Lumpur, MYS

**Keywords:** pediatric ophthalmology and strabismus, strabismus surgery, superior rectus rectraction, severe upshoot, duane retraction syndrome

## Abstract

Duane retraction syndrome (DRS) with mechanical and innervational upshoot poses a surgical challenge. We discuss a case of DRS with mechanical and innervational upshoot and its surgical management. An 11-year-old boy presented with left eye upward deviation since birth. This deviation was worst on the right gaze. His best corrected visual acuity was 6/6 OD and 6/60 OS. Refraction showed low hyperopia with low astigmatism in both eyes. Stereoacuity was absent and there was suppression on the Worth 4 dot test in the left eye. The left eye had large hypertropia of 50 prism diopter in primary gaze. Extraocular movements showed severe upshoot and narrowing of palpebral fissures on adduction and limited abduction (-2). The patient underwent Y-splitting of the left lateral rectus (LR) muscle of 10 mm, LR recession of 4 mm, and left eye superior rectus recession of 12 mm. A marked reduction in hypertropia in primary gaze was observed on day one and at two months postoperatively with residual upshoot on adduction. His left eye deviation remained stable after six months postoperatively.

## Introduction

Duane retraction syndrome (DRS) is a rare entity and its incidence in patients with strabismus is less than 5% [1]. Concomitant contraction of the lateral rectus (LR) muscle in adduction results in characteristic globe retraction with narrowing of the palpebral aperture and upshoot and/or downshoot in adduction. Abduction deficiency is frequently observed as agenesis of the abducent nucleus is a well-known theory for its underlying etiology [1]. The paradoxical synergistic innervation of the LR muscle by different nerves is evidenced by electromyographic (EMG) studies, and DRS has been further classified into Duane I, II, and III [2]. In 1988, Ahluwalia et al. further classified DRS according to the position of the affected eye: esotropia DRS, exotropia DRS, or orthotropic DRS [3].

The abnormal innervation may involve vertical muscles in the affected eye as demonstrated by an associated overshoot of the eye either over elevation or over depression, or in other words, upshoot or downshoot. These phenomena are reported in approximately 25-39% of patients with DRS [4]. The underlying mechanism for vertical movement abnormality can either be mechanical, innervational, or both [5]. Tight LR on the globe and co-innervation of the vertical or oblique muscles with the LR muscle are said to be the mechanisms involved. Our patient had a large vertical deviation in the primary gaze, a disfiguring overshoot occurred abruptly in adduction. This clinical finding is attributed to both mechanical and innervational pathology, which is a rare finding.

The surgical approach for our patient was directed toward (1) hypertropia in the primary gaze and (2) severe and disfiguring upshoot that highlighted the need for vertical muscle intervention. Thus, we report a rare case of DRS with disfiguring severe upshoot (mechanical and innervational-type) and its challenging surgical correction. We believe this case report will assist in decision-making in terms of planning for surgery for this rare type of DRS.

## Case presentation

**Figure 1:**
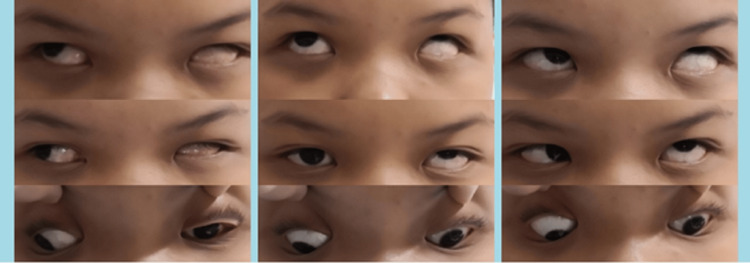
Preoperative extraocular movements in all gazes showing hypertropia of the left eye at primary hyperopia, severe upshoot on adduction with narrowing of the palpebral fissure, and limited abduction of the left eye

An 11-year-old boy presented with an upward deviation of the left eye since birth. This deviation was worst on the right gaze. On examination, his best corrected visual acuity was 6/6 OD and 6/60 OS. Refraction showed low hyperopia and astigmatism in both eyes. The left eye was hypertropic while the right eye was fixated on distant objects in primary gaze. Extraocular movements examination revealed evidence of globe retraction with narrowing of the palpebral fissure and severe upshoot on attempted adduction. On levoversion, the left eye had limited abduction (-2) and these findings are clinically well described in DRS type III (Figure 1).

The patient had left eye suppression on the Worth 4 dot test and absence of stereopsis upon testing with Titmus fly and Frisby tests. The prism cover test at the primary position showed incomitant left eye hypertropia of 50 PD base down and exotropia of 20 PD base in for distance at 6 meters and left eye hypertropia of 45 PD base down and exotropia of 18 PD base in at near. The forced duction test was positive for the superior rectus and tightness of both horizontal muscles in the left eye but was negative for the right eye in all directions. Fundus examination for both eyes was normal. The findings of the positive forced duction test and large left hypertropia in all gazes suggested the possibility of superior rectus contracture.

The patient underwent left eye superior rectus recession of 12 mm and 4 mm left LR recession with Y-splitting of LR muscle 10 mm from origin insertion. Superior rectus recession was done via the direct scleral technique. Traction suture Vicryl 5/0 was placed at the superior rectus muscle insertion to pull the globe inferiorly to achieve maximum exposure to enable visualization.

**Figure 2:**
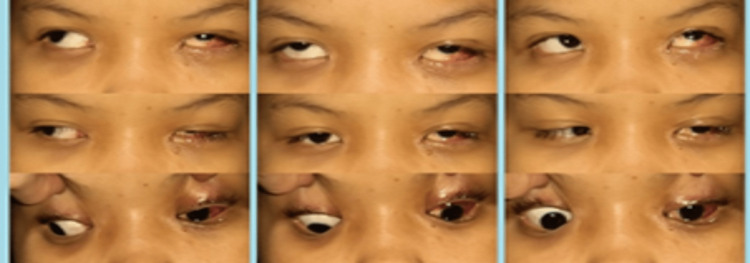
Postoperative day one: postoperative extraocular movements in all gazes following left superior rectus recession, lateral rectus recession, and Y-split showing a marked decrease of left hypertropia on primary gaze and severe upshoot on adduction

**Figure 3:**
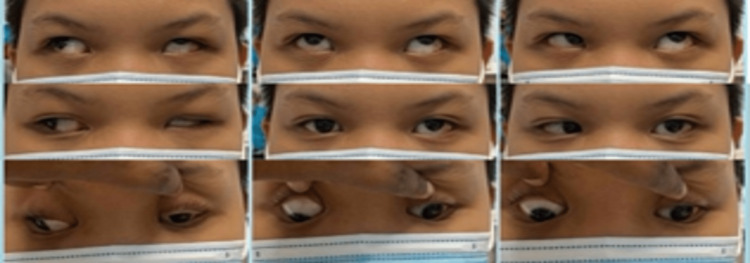
Extraocular movements two months after surgery showing marked reduction of hypertropia in primary gaze

Immediate postoperative examination on day one revealed the reduction of hypertropia of the left eye in primary gaze and levoversion (Figure 2). At the subsequent follow-up two months postoperatively, the residual hypertropia on primary gaze and on levoversion was stable with residual upshoot of the left eye on adduction despite a large 12 mm of superior rectus recession (Figure 3). The prism cover test showed 18 PD base down and 16 PD base in.

**Figure 4:**
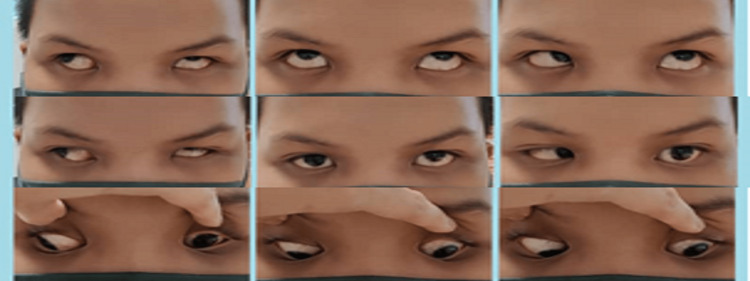
Images at six months postoperatively showing that left eye condition remained the same and prism cover test remained stable

Six months later, his left eye condition remained the same, and the prism cover test remained stable (Figure 4).

## Discussion

DRS is a complex and rare form of congenital strabismus; its incidence in patients with strabismus is less than 5% [6]. DRS is easily recognized clinically because it has a pronounced abduction deficit with varying degrees of adduction limitation, globe retraction with narrowing of the palpebral opening, and oblique elevation or depression on attempted adduction [6]. Despite the fact that the Huber classification is well-known, paradoxical contraction due to variable degrees of medial rectus (MR), inferior oblique (IO), and vertical rectus muscle innervation is often overlooked [2]. As a result, the term "atypical DRS" is used to describe clinical conditions that do not fit the Huber classification. Variable degrees of upshoot or downshoot movements on adduction and hypertropia in the primary position constitute the evidence for co-contraction of vertical rectus muscle in DRS, which Huber does not explain in his classification [7].

The superior rectus muscle's co-innervation with the LR muscle, or mechanical factors such as the bridle or leash effect created by tight LR, can cause an upshoot or downshoot [8]. When the globe adducts and moves above or below the horizontal plane, the tight LR slips, causing an upshoot or downshoot. In extreme circumstances, the "knife edge effect" has been characterized as manifesting even with the slightest adduction movement. Furthermore, a long-standing upshoot in the main position with substantial hypertropia may have led to the development of superior rectus contracture and subsequent fibrosis as demonstrated in our patient [8].

DRS is defined as a defect in nerve innervation of the LR muscle caused by the absence of the abducent nucleus or nerve, resulting in oculomotor nerve co-innervation and co-contraction [1]. Evidence from MRI studies and histological discoveries supports this postulation [9]. This result backs previous EMG investigations that showed a paradoxical contraction of the LR muscle during adduction [2]. In addition, DRS is inherited as an autosomal dominant trait in 10% of instances, and hence genetic factors play a part in its etiology [10].

To treat DRS type III, a variety of surgical approaches have been proposed. The recession of both the LR and MR of the affected eye can be used to treat unilateral DRS type III with upshoots or downshoots on adduction. Posterior fixation suture of the horizontal rectus, lowering of the insertion of the LR muscle, and vertical rectus recession are all surgical techniques used in DRS. Other techniques include LR recession with bifurcation (Y-split), in which the LR is split into two for 10 mm from the insertion and vertically transposed [10]. Secondary superior rectus contracture is treated with a large recession of the superior rectus muscle [11].

Arora et al. have documented a similar presentation in a 23-year-old female. Their team performed bilateral LR recession of 6 mm, Y-split of the LR, and superior rectus recession of 6 mm. The outcome at three months was good, with greatly reduced hypertropia [8]. In contrast, in our case, only unilateral LR recession and large superior rectus recession were performed as compared to bilateral LR recession and moderate superior rectus recession in the above-mentioned case. However, a good outcome was similarly observed in our patient.

Our patient had DRS with considerable upshoot, significant hypertropia in the primary position, and superior rectus contracture. The condition was successfully treated by using superior rectus recession, LR recession, and Y-splitting of the LR muscle. Our patient had residual hypertropia in the primary position after having achieved maximum superior rectus recession. One of the difficulties in managing DRS is the associated superior rectus contracture [12].

## Conclusions

DRS can be linked to the adduction upshoot or downshoot of the affected eye. Hypertropia on primary gaze in DRS is rare. Clinicians should be aware of the likelihood of executing a combination of surgical techniques and handle their cases accordingly.

## References

[1] DeRespinis PA, Caputo AR, Wagner RS, Guo S (1993). Duane's retraction syndrome. Surv Ophthalmol.

[2] Huber A (1974). Electrophysiology of the retraction syndromes. Br J Ophthalmol.

[3] Ahluwalia BK, Gupta NC, Goel SR, Khurana AK (1988). Study of Duane's retraction syndrome. Acta Ophthalmol (Copenh).

[4] Isenberg S, Urist MJ (1977). Clinical observations in 101 consecutive patients with Duane's retraction syndrome. Am J Ophthalmol.

[5] Kraft SP (1988). A surgical approach for Duane syndrome. J Pediatr Ophthalmol Strabismus.

[6] Duane A (1996). Congenital deficiency of abduction, associated with impairment of adduction, retraction movements, contraction of the palpebral fissure and oblique movements of the eye. 1905. Arch Ophthalmol.

[7] Özkan SB (2017). Pearls and pitfalls in the management of Duane syndrome. Taiwan J Ophthalmol.

[8] Arora P, Ganesh S, Shanker V (2014). Duane′s retraction syndrome with severe upshoot and ipsilateral superior rectus contracture: a rare presentation. J Clin Ophthalmol Res.

[9] Kang NY, Demer JL (2006). Comparison of orbital magnetic resonance imaging in Duane syndrome and abducens palsy. Am J Ophthalmol.

[10] Kekunnaya R, Negalur M (2017). Duane retraction syndrome: causes, effects and management strategies. Clin Ophthalmol.

[11] Mohan K, Saroha V (2002). Vertical rectus recession for the innervational upshoot and downshoot in Duane's retraction syndrome. J Pediatr Ophthalmol Strabismus.

[12] Akbari MR, Manouchehri V, Mirmohammadsadeghi A (2017). Surgical treatment of Duane retraction syndrome. J Curr Ophthalmol.

